# Lessons learned from implementation of a demonstration program to reduce the burden of anemia and hookworm in women in Yen Bai Province, Viet Nam

**DOI:** 10.1186/1471-2458-9-266

**Published:** 2009-07-28

**Authors:** Tran Q Phuc, Seema Mihrshahi, Gerard J Casey, Luong B Phu, Nong T Tien, Sonia R Caruana, Tran D Thach, Antonio Montresor, Beverley-Ann Biggs

**Affiliations:** 1 National Institute of Malariology, Parasitology and Entomology (NIMPE), Hanoi, Viet Nam; 2 Department of Medicine (RMH/WH), The University of Melbourne, The Royal Melbourne Hospital, Parkville, Victoria, 3050, Australia; 3 Yen Bai Centre of Malariology, Parasitology and Entomology, 395 Yen Ninh St Yen Bai, Viet Nam; 4 Research And Training Centre for Community Development, 39 Lane 255, Vong St Hanoi, Viet Nam; 5 World Health Organization, 61 Tran Hung Dau St Hanoi, Viet Nam; 6 Centre for Clinical Research Excellence in Infectious Diseases (CCREID), The Royal Melbourne Hospital, Parkville, Victoria, 3050, Australia

## Abstract

**Background:**

Iron deficiency, anemia and hookworm disease are important public health problems for women of reproductive age living in developing countries and affect the health of newborns and infants. Iron supplementation and deworming treatment are effective in addressing these problems in both pregnant and non-pregnant women. Daily iron supplementation and deworming after the first trimester is recommended for pregnant women although these programs usually do not operate efficiently or effectively. Weekly iron-folic acid supplementation and regular deworming for non-pregnant women may be a viable approach for improving iron status and preventing anemia during the reproductive years. Addressing these diseases at a population level before women become pregnant could significantly improve women's health before and during pregnancy, as well as their infants' growth and development.

**Methods and Results:**

This paper describes the major processes undertaken in a demonstration intervention of preventive weekly iron-folic acid supplementation with regular deworming for all 52,000 women aged 15–45 years in two districts of Yen Bai province, in northern Viet Nam. The intervention strategy included extensive consultation with community leaders and village, commune, district and provincial health staff, and training for village health workers. Distribution of the drugs was integrated with the existing health service infrastructure and the village health workers were the direct point of contact with women. Iron-folic acid tablets and deworming treatment were provided free of charge from May 2006. An independent Vietnamese NGO was commissioned to evaluate compliance and identify potential problems. The program resulted in effective distribution of iron-folic acid tablets and deworming treatment to all villages in the target districts, with full or partial compliance of 85%.

**Conclusion:**

Training for health staff, the strong commitment of all partners and the use of appropriate educational materials led to broad support for weekly iron-folic acid supplementation and high participation in the regular deworming days. In March 2008 the program was expanded to all districts in the province, a target population of approximately 250,000 WRA, and management was handed over to provincial authorities.

## Background

Iron deficiency and anemia are important public health problems for women of reproductive age (WRA) living in developing countries. Iron deficiency has been estimated to cause 30–70% of all maternal anemia [1]. Hookworm infection is a significant predictor of poor iron status and iron deficiency anemia in pregnant and lactating women, and is also a disease of public health importance globally. Iron deficiency anemia is an important factor in susceptibility to infectious disease [2], decreased work capacity [3,4], and maternal death [5]. Maternal anemia may have an effect on preterm birth [6] and has been shown to be a significant predictor of low birth weight [7-9] and iron deficiency in infants, which has negative effects on child development [10,11].

### Interventions to control anemia and hookworm infections

The main interventions that have been implemented to address iron deficiency anemia include iron supplementation, iron fortification of foods and nutrition education programs [1]. The World Health Organization (WHO) recommends routine daily iron supplementation during pregnancy [12]. However, few countries have reported improvement in anemia rates at a national level and, generally, the effects of large scale daily iron supplementation in pregnancy have been disappointing due to poor compliance [13-15], cost [16], side effects [17,18], and lack of education about the importance of iron in pregnancy [19].

Recent evidence suggests that weekly iron-folic acid supplementation (WIFS) is as effective as daily supplementation for reducing the burden of iron deficiency anemia in non-pregnant WRA [20-24], and WHO recently released a position statement recommending WIFS for WRA in areas where the prevalence of anemia is above 20% [25]. However, there are few studies reporting the operational considerations in large-scale community-based WIFS programs [26,27].

This paper will describe the lessons learned from the implementation of a preventive WIFS and deworming program for WRA living in Yen Bai province, northern Viet Nam, and summarizes the experiences and factors that should be considered in future programs. The main aims of the program were to measure the prevalence of anemia and hookworm infection in WRA; implement a demonstration WIFS/deworming intervention for WRA in order to reduce the prevalence of iron deficiency anemia and hookworm infection in this group; and to provide evidence of the feasibility, effectiveness, compliance, sustainability and cost of the program.

## Methods and results

### Project design

#### 1. Project site

Yen Bai province was chosen as the project site because it was identified as a poor rural province that was expected to have high rates of anemia and hookworm infection. The province of Yen Bai consists of nine administrative towns and districts. Governance in the province is by provincial civil committees (People's Committee, Communist Party Committee) and service departments (e.g. Department of Health Services etc), through their district and commune counterparts, to the village and hamlet level.

#### 2. Situation analysis and formative research

Project staff visited Yen Bai province in April 2005 to undertake a situation analysis and collect background information from key informants that could be used to design an initial survey to measure the prevalence of anemia, iron deficiency and hookworm infection in WRA (defined by the Yen Bai Health Service as women aged 16–45 years). The Yen Bai Centre for Malariology, Parasitology and Entomology (CMPE) was identified as an appropriate provincial project partner.

Meetings were convened with health officials, clinicians, teachers, members of community organizations and the general community to discuss a WIFS/deworming intervention and address logistic considerations, such as the operational and human capacity to implement the intervention. Other information gathered included the demographic characteristics of the population, presence of existing infrastructure, details of other local and international groups working in Yen Bai, and the geography and transport routes of the province. National policies regarding prevention and control of intestinal parasite infections and prevention and control of iron deficiency and/or anemia were also reviewed.

In November 2005 a baseline survey of anemia and hookworm prevalence was undertaken in Tran Yen and Yen Binh districts [28]. Demographic and socio-economic data from this survey is presented in Table 1. The prevalence of anemia (Hb<120 g/L) was 37.5% (131/349) and the prevalence of iron deficiency (ferritin <15 μ g/L) was 23.1% (81/349). Hookworm infection was present in 78.2% (261/334) of women. On the basis of these results, it was decided to proceed with a WIFS/deworming intervention that targeted approximately 52,000 WRA in two districts, and if successful to scale up to a province-wide program. For this and all other surveys informed consent was documented prior to enrolment.

**Table 1:** Demographic and socioeconomic data for WRA in study area, November 2005, Yen Bai Province, Viet Nam. (N = 382*)

Variable		N (%)
Mean Age (95% CI)		31.73 (30.93–32.53)

Marital status	Married	320 (85%)
	Not married	54 (14%)
	Divorced/Widow	3 (<1%)

Number of children	Median (range)	2 (0–5)

Ethnicity	Kinh	252 (66%)
	Tay	46 (12%)
	Cao Lan	29 (8%)
	Dao	50 (13%)
	Other	3 (<1%)

Education level	Illiterate	27 (7%)
	To Grade 5	57 (15%)
	Grade 6–9	210 (56%)
	Grade 10–12	70 (19%)
	Higher	10 (3%)

Current pregnancy status	Pregnant	6 (2%)
	Not pregnant	370 (98%)

Frequency of wearing shoes	Never	44 (12%)
	Occasionally	184 (50%)
	Always	142 (38%)

* Failure to sum to 382 indicates the woman's uncertainty or no response.

Tran Yen and Yen Binh districts were chosen for the intervention as they were easy to reach, and had a representative population of Kinh and ethnic minority groups. It was decided that the supplements would have to be actively supplied and free of charge in order to be equitable for poor families, for ease of administration and to aid compliance. Provincial and district health staff recommended that village health workers (VHWs) play a key role in delivery of deworming and iron supplements to WRA because they had direct access to women in their communities and could work with the Women's Union to mobilize the target group and support project and health promotion activities.

#### 3. Project administration and funding

The University of Melbourne (UoM) and the Walter and Eliza Hall Institute of Medical Research (WEHI) were the international partners; the Viet Nam National Institute for Mariology, Parasitology and Entomology (NIMPE) the national implementing agency; the Yen Bai CMPE the provincial implementing agency; and WHO Hanoi provided technical advice. During the late planning stages it became apparent that implementation and monitoring would benefit from a Viet Nam-based project manager. WHO Western Pacific Regional Office gave advice regarding the WIFS component of the program. Funding was obtained from Atlantic Philanthropies (USA) Inc.

### Implementation of the program

**Figure 1:**
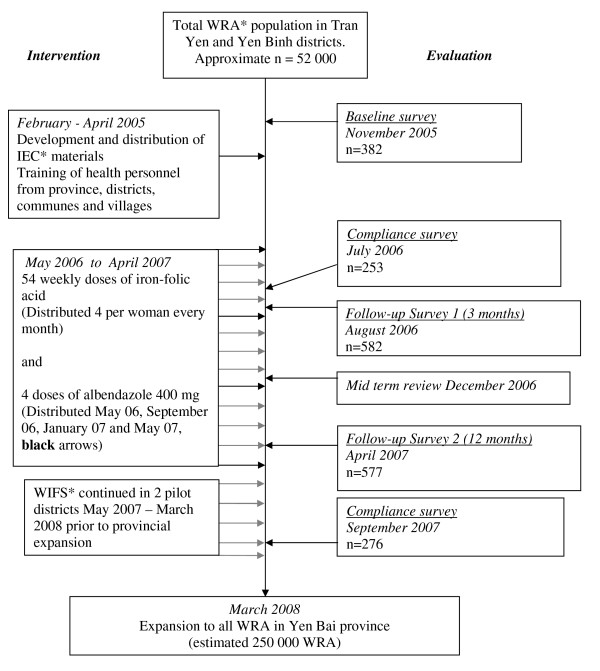
**Design and evaluation of a preventive WIFS and deworming program**. *WRA = Women of Reproductive Age defined as 16 – 45 years. IEC = Information, Education and Communication. WIFS = Weekly Iron-Folic acid Supplementation.

The project was implemented in stages as shown in Figure 1 after further meetings with the provincial People's Committee and health services and district and commune health staff to discuss the purpose and methodology of the intervention.

#### 1. Advocacy and IEC materials

**Figure 2:**
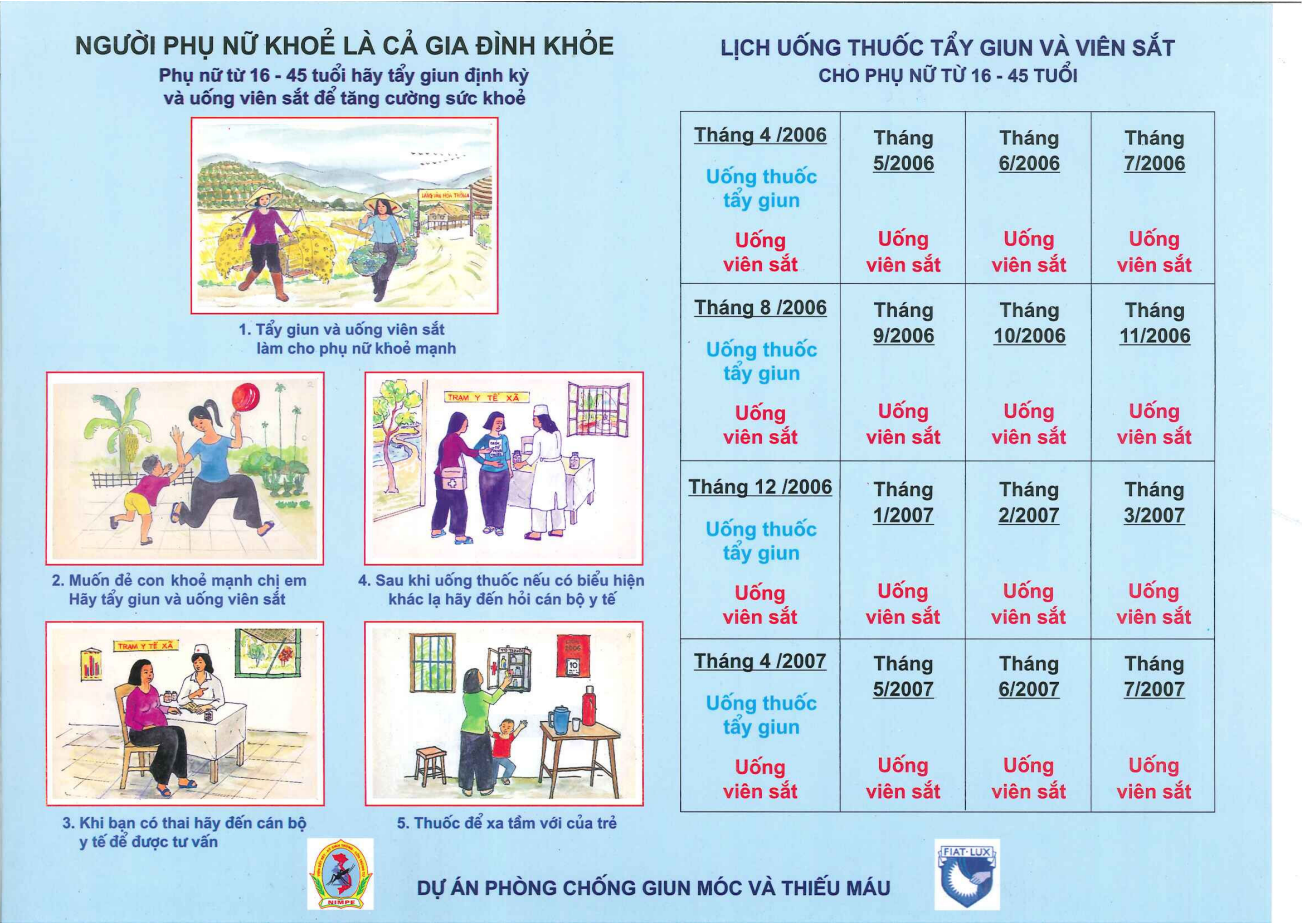
**First promotional handout for women, April/May 2006**.

Promotional materials were developed in conjunction with commune health staff and VHW and included posters for health staff (4 copies for every village and commune) and a pictorial handout with timetable for all WRA (Figure 2). These were field tested at selected sites prior to implementation.

The main messages that were communicated to the women on the handout were:

"Healthy Women – Healthy Families"

▫ All women aged 16–45 should take deworming and iron to ensure good health

▫ Deworming and iron help women to feel and work better

▫ Take deworming and iron to provide a healthy happy environment for children

▫ If you think you are pregnant go to commune health station

▫ If you feel sick go to the commune health station

▫ Keep all tablets away from children

In addition, banners were produced at all commune health stations for the launch of the intervention. Provincial television coverage including interviews with major partners was also organised.

#### 2. Training and incentives for health staff

Before the intervention was implemented, a one week Training of Trainers session was conducted by experienced NIMPE trainers for four CMPE staff. These staff then conducted two day training sessions for four staff of each district Centre for Preventive Medicine, two nurses from each commune health station and all VHW in the two districts, a total of 680 health personnel. The training focused on the causes, health risks, treatment, and prevention of anemia and hookworm infection: use of the IEC materials; effective IEC dissemination skills: distribution strategy: reporting requirements and responsibilities: monitoring, reporting and responding to adverse events: and monthly follow up of women. All staff (including VHW, commune and district staff) involved in the project received a small monthly stipend during the implementation period.

#### 3. Distribution strategy

A bulk supply of iron-folic acid tablets was procured from UNICEF Copenhagen. NIMPE was responsible for ensuring timely transfer to the province. In the province the tablets were stored centrally at the CMPE. Population estimates were taken from the Yen Bai Statistics Handbook, 2004 [29] and the number of women targeted was 52,000.

The transfer of tablets to each district occurred during the approximately monthly visits by the districts' Centres of Preventive Medicine personnel to the CMPE for supplies and administrative requirements, to the commune during the monthly meeting attended by commune health station staff held at the Centre of Preventive Medicine in all districts, and by commune staff to VHWs. VHWs then distributed the tablets on a monthly basis to the WRA in their village.

Reporting at each stage included number of tablets received, number disbursed, number of women accessing the service and, from the commune, adverse events.

#### 4. Intervention

The implementation period for the demonstration project was from May 2006 to April 2007. No other interventions were initiated in Tran Yen or Yen Binh between November 2005, when the baseline survey was conducted, and May 2006 when the WIFS/deworming intervention commenced. WIFS/deworming was continued in the two districts from May 2007 to March 2008 when a province-wide expansion was implemented.

##### Iron tablets

Free monthly supply of ferrous sulphate/folic acid tablets (200 mg, equal to 60 mg elemental iron/0.4 mg folic acid, UNICEF, Copenhagen) to be taken weekly was given directly to WRA by VHW. The tablets were coated, brown/red in colour and were supplied in double-lidded tamper proof bottles to the women (i.e. 4 tablets per bottle per month). The women were strongly instructed to keep the bottles out of reach of children and the bottles were also labeled with this information. To aid compliance, women were encouraged by the VHWs to take the iron-folic acid tablet on the same day each week and were given instructions about swallowing the tablets with water, not taking them on an empty stomach, and also avoiding tea/coffee at the time of taking the tablets.

The Viet Nam Ministry of Health guidelines recommend pregnant women take one iron-folic acid tablet (120 mg/0.4 mg) daily, distributed through commune health stations. In the communes where this program was operational, women who became pregnant were advised to access this service instead of taking the WIFS; however it was not universally available. In areas where it was not available, pregnant women were provided with an additional weekly ferrous sulphate tablet (60 mg elemental iron, UNICEF, Copenhagen), to be taken from the second trimester until three months post-partum.

##### Deworming

Albendazole (400 mg, UNICEF, Copenhagen) was administered to non-pregnant women as observed treatment on locally designated days either at the commune health station or supervised in the village by a commune health worker. Pregnant women were identified by asking women whether they were pregnant and the timing of their last menstrual period. In this way, we were able to follow government guidelines, which avoid giving deworming treatment to pregnant women. The tablets were supplied in tins of 100. Tablets were non-coated and white in colour. Women were observed for 30 minutes at the health station after administration of the tablets to ensure vomiting did not prevent consumption of the tablet.

#### 5. Monitoring and Evaluation

##### (i) Compliance monitoring

The Research and Training Centre for Community Development (RTCCD) was commissioned to evaluate compliance in July 2006, 3 months after commencement of the intervention, and again in September 2007. A stratified multi-stage cluster sampling design was used. Primary sampling units (villages) were chosen using a 'probability proportional to size' random sampling method. Secondary sampling units (individual women) were selected randomly from each village. In the first survey 253 women were interviewed using a structured questionnaire, and in the second survey 276 women were interviewed. The surveys also reported that there were no interruptions in supply of tablets over the period of the intervention.

##### (ii) Anemia, iron deficiency and hookworm prevalence surveys

The same stratified multi-stage cluster sampling design for choosing women for the baseline survey was used for the three (August 2006, n = 338) and 12 (April 2007, n = 364)-month follow-up surveys. Although we were mainly interested in the population effect of the intervention, women who took part in the baseline survey were also invited to participate in follow-up surveys in August 2006 (n = 244) and April 2007 (n = 213) to allow monitoring of individual responses, and to compare the outcome variables (Hb, ferritin, and parasite infection) between subjects included in the baseline survey with those who were not, to allow for the possibility that inclusion in the baseline survey might affect compliance with the intervention and hence response to treatment. All surveys were conducted by the same teams and included trained phlebotomists, stool preparation and analysis technicians, a demographic recorder and a supervisor. Sample collection and laboratory analysis has been previously reported [28] and was the same in each of the surveys.

##### (iii) Survey results

The results of monitoring and evaluation surveys will be published elsewhere. In summary, independent monitoring using structured questionnaires identified that full compliance with iron-folic acid tablets (taking all tablets as scheduled) was 69% (191/276) and partial compliance (taking some but not all tablets) a further 16% (43/276) at the end of demonstration project. The laboratory-based survey after twelve months identified that anemia prevalence had fallen from 37.5% to 19.5% (111/569) and hookworm prevalence from 78.2% to 25.1% (117/467).

#### 6. Feedback and project responses

Monthly trips to district centres, communes and villages were undertaken by the project team to identify problems and areas in need of more support. Formal and informal meetings were held with provincial Women's Union leaders, commune Communist Party heads and village leaders and presentations were made to outline the impact of the project and to ensure continued support.

One of the recommendations from the first compliance survey in July 2006 was that stronger project promotion was required in mountainous communes with high proportions of ethnic minority groups. In response, a calendar was produced for all women in the project districts. This displayed photos of healthy women from various ethnic minority groups with healthy babies and photos of healthy foods. In addition an iron-folic acid tablet was depicted on every Sunday as a memory aid.

VHWs requested a more comprehensive collection of educational materials to use in IEC activities. The materials developed included a set of nine posters, five for hookworm and four for anemia, with photos and simple messages on one side and questions and answers for discussion on the reverse side. The messages placed more emphasis on the causes and health consequences of anemia and hookworm infection for both women and newborns and the benefits to both of treating and controlling these diseases. Further information was made available for broadcasting over the village loudspeaker system.

Considerable confusion about the extra iron tablet for pregnant women was also identified. Women were unsure about whether to access both project and government iron or only one source. In response, the message was reinforced that women should take the government daily supplement where available, and if not available, they should access the extra weekly tablet through the WIFS program.

The use of plastic bottles was also a problem for both VHWs and WRA. For the VHW, receiving bulk tablets and having to distribute them was inconvenient and inefficient. Women found that the bottle was easy to forget or misplace and an unacceptable number of tablets were spoiled. To overcome these issues subsequent iron-folic acid tablets for the expansion were sourced from a Vietnamese pharmaceutical manufacturer (Naphaco, Nam Dinh) and packaged in purposely designed modified blister packs. These packs contained six tear-off strips each with five tablets.

#### 7. Mid-term project review meeting

A one day workshop was organized by the project partners in conjunction with the Yen Bai People's Committee and Yen Bai Health Services. The meeting was held in Yen Bai city in December 2006. Participants included representatives from district health authorities and People's Committees, Women's Union, provincial and district hospitals as well as members of the project team from NIMPE, RTCCD, UOM and WHO.

#### 8. Ethics

The project was approved by the Human Research Ethics Committee of the National Institute of Malariology, Parasitology and Entomology (Hanoi, Vietnam), the Walter and Eliza Hall Institute of Medical Research (Melbourne, Australia) and Melbourne Health (Melbourne, Australia).

## Discussion

The most important underlying concept in the development of the WIFS/deworming treatment project was that it would eventually become sustainable and have broad community support. Therefore, the design included wide-ranging, open discussions and involvement of all partners from international to local level. As a result everyone understood the goals, benefits and underlying principles of the project, and had the flexibility needed to adapt to particular local conditions in order to achieve these goals.

Table 2 summarizes the main lessons learned. An important consideration was that VHWs received training that included health information and IEC skill development, as they were the critical point of contact with WRA. A second consideration was that the IEC program needed to provide information about anemia and soil-transmitted helminths (STH) and their consequences, treatment and control in an easily understood format.

**Table 2:** Main factors for consideration in future preventive programs to reduce the burden of anemia and hookworm in women

Activity	Output
Identify appropriate local partners	The Yen Bai CMPE was able to advise the national and international partners on local issues and facilitate communication between the important provincial authorities and all levels of the provincial health service structure.

Conduct informative situation analysis	Formative research and a valid needs assessment for the project was conducted which identified that anemia and hookworm disease were major health problems for the community as well as health authorities. This was vital to providing the program with clear purpose, helped with the development of goals and gave the program credibility

Develop plan collaboratively	Open discussions between all parties ensured the program design would be accepted and supported by all involved.

Maintain communication	Regular meetings between the project team and local parties ensured that results were communicated to the province in a timely manner and potential problems could be resolved before they influenced outcomes.

Appropriate point of contact for target group	Village health workers were identified as the most appropriate point of contact because they lived in the communities and were well respected among the target group. VHW would also feel ownership and responsibility for the program and thereby help with sustainability

Appropriate, adaptable training materials developed and assessed	Culturally appropriate training and IEC materials ensured VHW had sufficient knowledge of the health problems being addressed and the resources to communicate that knowledge to WRA.

Packaging of tablets	With a small increase in cost a more acceptable form of packaging was developed.

Independent monitoring of the intervention conducted early in the process	The survey conducted early (at three months) in the intervention resulted in modification of the training, materials and repackaging of the tablets.

Intervention supplied free of charge	This helped with equitable distribution and helped successful implementation of the intervention

Incentives for staff	Small payments and feedback to staff about the project results were given to staff during the course of the program. These helped the implementation of the program

Impact evaluation	Evaluation surveys to document impact have provided project partners with data and information for advocacy with stakeholders and policy makers, and dissemination of results to the international community.

In Viet Nam the health system generally enjoys a strong vertical structure from the provincial level down to the village. Hence, successfully integrating the introduction of iron-folic acid tablets and the administrative monitoring process was a matter of making small modifications to the existing system. This process may require more attention in countries where the health service structure is weaker. In Viet Nam the critical factors for the successful integration of this program were, i) the active support of the relevant political and health authorities, ii) a clear understanding by all parties of the health issues and how the program addressed those issues, iii) a simple distribution process and iv) active provision of WIFS/deworming to WRA.

Monitoring and evaluation of the project were conducted at two levels. The impact on women's iron status and worm burden was assessed through follow-up laboratory surveys conducted as part of the project. Independent monitoring by RTCCD was utilized to assess compliance, acceptability, promotional effectiveness and general day to day management issues. The three month compliance survey was useful because it identified intrinsic problems that needed to be addressed for the long term success of the program. We used the sixteen month (September 2007) survey to assess compliance as well as the likely impact of handing over the program to the province, and to identify areas that would need assistance prior to the external collaborators withdrawal from regular management. One of the findings of this survey was that women liked the colourful calendars but they did not have a great effect on compliance. The community education meetings were a greater factor in maintaining high compliance. Repackaging of tablets in a modified blister pack was also greatly appreciated by both VHWs and the women.

A guiding principle of the project was that the supplements be provided free of charge to all WRA. This ensured that there was equitable distribution to poorer women especially ethnic minority groups. It also avoided a complicated system of cost recovery and helped achieve high compliance levels.

In our project, women were asked if they were pregnant at each deworming event and if so advised not to take the treatment in accordance with Vietnamese guidelines. WHO evaluated publicly available and confidential reports of more than 6000 women that had *inadvertently *taken mebendazole or albendazole during pregnancy and concluded that the use of anthelminthic drugs during pregnancy does not increase the risk of harm to the foetus. They recommend that single dose oral anthelminthic can be given to pregnant and lactating women but should be avoided in the first trimester [30]. Reassuringly, studies in Sri Lanka [31], Sierra Leone [32] and Nepal [33] have consistently found that anthelminthic treatment during pregnancy significantly improved iron status and increased newborn body weight. Despite this clarification from WHO, several countries (including Viet Nam) are still reluctant to administer anthelminthic therapy at any stage during pregnancy. In order to reduce the potential for inadvertent treatment of women in the first trimester of pregnancy participating in mass deworming programs the questions 'Are you pregnant?' and 'When was your last menstrual period?' can be used to screen for pregnancy and should be asked prior to administration [30,34]. However, studies on the effectiveness of these questions as screening tools have not been reported. Further research on this issue would be of great benefit to future programs. The other issue for program managers is to consider follow-up procedures for women who have deworming treatment withheld during pregnancy, so that these women have access to treatment at the earliest possible time.

WIFS/deworming treatment is now available to women throughout Yen Bai province. The ownership of the project and training of VHWs has been transferred to the provincial and district authorities and their communities. Delivery of Vietnamese-produced iron-folic acid supplements to Yen Bai has become part of the manufacturing contract ensuring timely delivery. Albendazole is provided by WHO as part of their ongoing program of deworming in Viet Nam. In 2006, it was estimated that the approximate price for weekly iron-folic acid tablets and four monthly deworming was USD0.20 per women per year based on UNICEF bulk supplied iron-folic acid tablets (personal communication D. Satori, unpublished results). Adjusted for locally produced, blister packed tablets, this cost is now approximately USD0.30 per woman per year (2009 pricing).

A major challenge is to maintain the current high level of participation and enthusiasm as the project is integrated into the routine provincial health program. This is most likely to be achieved if WRA perceive a health benefit from taking weekly iron and regular deworming. To assist this, survey results have been communicated to commune and village health workers to pass on to their communities. Women's support will provide upwards pressure on local health authorities to maintain the program long term. During the demonstration project, VHWs, commune and district staff in the two districts received a small monthly payment from project funds. This was discontinued when the program expanded to the entire province, and provincial authorities have since made their own arrangements for a small sustainable payment at the time of biannual deworming.

## Conclusion

In conclusion, the strong commitment of all partners to the intervention has led to excellent participation levels and a significant clinical impact. The program has been expanded province-wide reaching an estimated 250,000 WRA. As well as improving the overall health of women, this will facilitate women entering pregnancy with adequate iron stores, thereby improving pregnancy outcomes, with enhanced potential for healthy growth and development of infants in the crucial early years.

## Competing interests

The authors declare that they have no competing interests.

## Authors' contributions

GC, TP, SC, AM and B-AB developed the project protocol; GC, SC and B-AB provided project management; GC, SC, TP, and NT coordinated the project; TT coordinated the RTCCD surveys; GC and TP were responsible for managing the study teams; collecting data and data entry. SM wrote drafts of the manuscript with input from TP, GC and B-AB. All authors reviewed and contributed to drafts of the paper.

## Pre-publication history

The pre-publication history for this paper can be accessed here:


